# Beware of thermal epiglottis! A case report describing ‘teapot syndrome’

**DOI:** 10.1186/s12871-018-0665-7

**Published:** 2018-12-22

**Authors:** V. Verhees, N. Ketharanathan, I. M. M. H. Oen, M. G. A. Baartmans, J. S. H. A. Koopman

**Affiliations:** 1 Department of Intensive Care, Maasstad Ziekenhuis, Maasstadweg 21, 3079 DZ Rotterdam, the Netherlands; 2 Department of Pediatric Intensive Care and Department of Pediatric Surgery, Erasmus MC, Doctor Molewaterplein 40, 3015 GD Rotterdam, the Netherlands; 3 Burn Centre, Maasstad Ziekenhuis, Maasstadweg 21, 3079 DZ Rotterdam, the Netherlands; 4 Department of Paediatrics, Maasstad Ziekenhuis, Maasstadweg 21, 3079 DZ Rotterdam, the Netherlands; 5 Department of Anaesthesiology, Maasstad Ziekenhuis, Maasstadweg 21, 3079 DZ Rotterdam, the Netherlands

**Keywords:** Thermal epiglottitis, Airway injury, Burn injury

## Abstract

**Background:**

The type of scalding injury known as ‘teapot syndrome’, where hot liquid is grabbed by the child with the aim of ingestion and falls over a child causing burns on the face, upper thorax and arms, is known to cause peri-oral and facial oedema. Thermal epiglottitis following scalds to face, neck and thorax is rare and can occur even in absence of ingestion of a damaging agent or intraoral burns, Awareness of the possibility of thermal epiglottitis, also in scald burns, is imperative to ensure prompt airway protection.

**Case presentation:**

We report the case of a child with thermal epiglottitis after a scalding burn from boiling milk resulting in mixed deep burns of the face, neck and chest, but no history of ingestion. Upon presentation there was a progressive stridor and signs of respiratory distress requiring intubation. Laryngoscopy revealed epiglottis oedema, confirming the diagnosis of thermal epiglottitis. Final extubation took place 5 days after initial burn.

**Conclusions:**

Thermal epiglottitis following scalds to face, neck and thorax is rare and can occur even in absence of ingestion and intra-oral damage. Burns to the peri-oral area should raise suspicion of additional damage to oral cavity and supraglottic structures, even in absence of intra-oral injury or initial respiratory distress. Awareness of the occurrence of thermal epiglottitis in absence of intra-oral injury is important to diagnose impending upper airway obstruction requiring intubation.

## Background

Epiglottitis is characterised by inflammation and oedema of the epiglottis and adjacent tissue, which can rapidly develop into life-threatening upper airway obstruction. It requires quick diagnosis and medical intervention to protect a patent airway. Clinically, swelling of the epiglottis results in drooling, inspiratory stridor and signs of respiratory distress. Additional symptomatology can be related to aetiology. Traditionally, epiglottitis in children is caused by an infection with Haemophilus Influenzae. As a result of widespread Hib vaccination, the incidence of epiglottitis in children has fallen [1]. Reported non-infectious causes of epiglottitis include thermal injury, corrosive agents and foreign body ingestion [2–5]. Incidents of burns after ingestion of hot beverages, food or objects resulting in injury of the respiratory tract have been reported [3, 4]. It is unusual for scald burns to be accompanied by upper airway damage and obstruction, but it has been emphasised in case-reports as a complication in the presence of intra-oral damage [5, 7].

We report a case of unexpected thermal epiglottitis after a scald burn with boiling milk resulting in deep burns on face, neck, chest, arm and foot, because there was no history of hot milk ingestion or intra-oral damage. The absence of intra-oral damage in our case makes this a unique case report. Written informed consent was obtained from a parent. The case report was written following the CARE guidelines [6].

## Case description

**Fig. 1:**
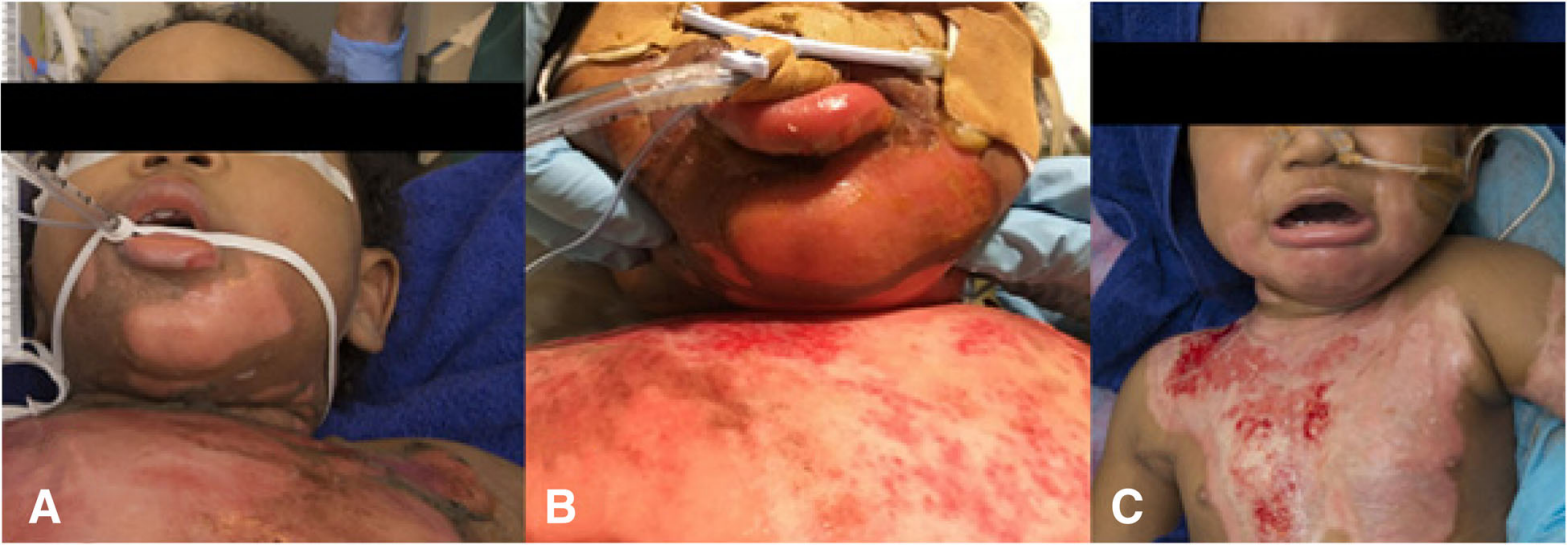
**a** Injuries upon presentation; **b** development of oedema 48 h post-burn; **c** inspection of injuries 21 days post-burn

A 15-month old boy, without significant medical history, was presented at the Burn Centre after a scald burn from hot milk, with mixed deep second degree burns to lips and chin, neck, chest, left arm and left foot covering 12% total body surface area (TBSA; burns to neck (1%); chest (4%); face (3%); foot (1%); left arm 3%) as assessed by palmar method. The mechanism of injury was submersion by just-boiled milk falling off the table, after he pulled the tablecloth the mug was standing on. There was no history of ingestion and no scalding or swelling of tongue or nostrils. He was cooled at the place of injury and assessed by the Helicopter Emergency Medical Services (HEMS). Intravenous rehydration according to Parkland formula (lactated ringers: 4 ml/kg/% TBSA of which half in the first 8 h, the remaining volume in the subsequent 16 h, with a maintenance of 2 ml/kg/h NaCl 0.9%/glucose 5%) was initiated and intravenous analgesics were given (fentanyl 1.5 mcg/kg and paracetamol 15 mg/kg). He had a progressive stridor with laboured breathing which the HEMS-physician ascribed to sputum stasis. With supplemental oxygen (non-rebreathing mask 12 L/min) his oxygen saturation was more than 95%. Hence he was considered medically stable during transport by the HEMS-physician. Upon first presentation at the Burn Centre he had deteriorated, his oxygen saturation was 80% despite maximal supplemental oxygen via a non-rebreathing mask. Inspection showed blistering off the lower lip concomitant with his burn injuries, without intra-oral redness or swelling (Fig. 1).

His circulation was not compromised. Despite airway opening manoeuvres and sputum evacuation, the stridor persisted. Due to persistent respiratory distress the decision was made to intubate. Pre-oxygenation took place by non-rebreathing bag (already in situ) and bag-mask ventilation after induction. Due to vomiting a rapid sequence induction was performed. Induction of anaesthesia was obtained with propofol 2.5 mg/kg, ketamine 1 mg/kg, fentanyl 3 mcg/kg and rocuroniumbromide 1 mg/kg. Bag-mask ventilation proved impossible due to airway obstruction. Classic airway opening manoeuvres and a guedell were ineffective. Direct laryngoscopy revealed a considerably enlarged and oedematous epiglottis without visible vocal cords. Blind intubation with a cuffed endotracheal tube (4,5 mm) was successful with help of a malleable stylet. Afterwards, ventilation was achieved with low pressure and bilateral air entry. The algorithm for difficult airway management would have been pursued in case of intubation failure by attempting videolaryngoscopy, emergency needle cricothyrotomy or a surgical airway. This algorithm was discussed with the entire emergency team prior to first intubation. All equipment, as well as expertise, necessary for a management of a difficult airway is present in the emergency room of the Burn Centre. After securing the airway, the burn injuries were debrided and dressed in silver sulfadiazine gauze. Rehydration in accordance with Parkland formula was maintained. Subsequently, he was transported to a paediatric intensive care unit in an academic paediatric hospital for continuation of mechanical ventilation.

The otorhinolaryngologist performed a direct laryngoscopy, demonstrating a fibrinous coating around the evidently oedematous epiglottis.

**Fig. 2:**
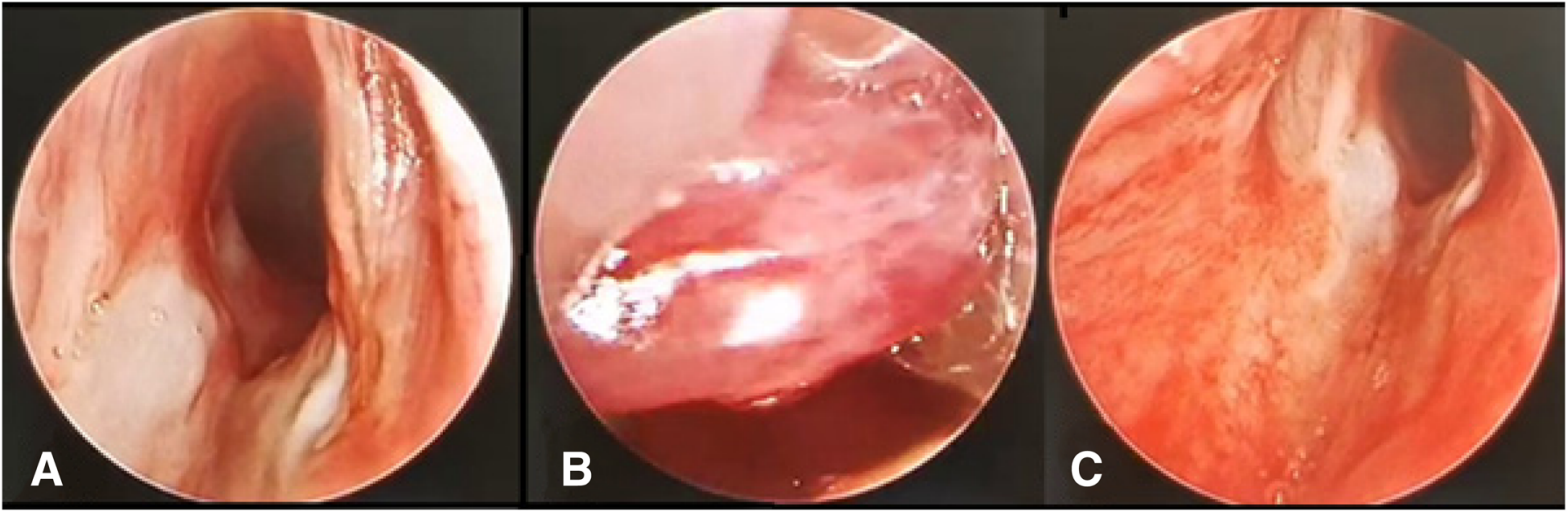
Fiberscopy 3 days post-burn after self-extubation showing an (**a**): airway with fibrinous coating; (**b**) oedematous epiglottis and (**c**): airway

**Fig. 3:**
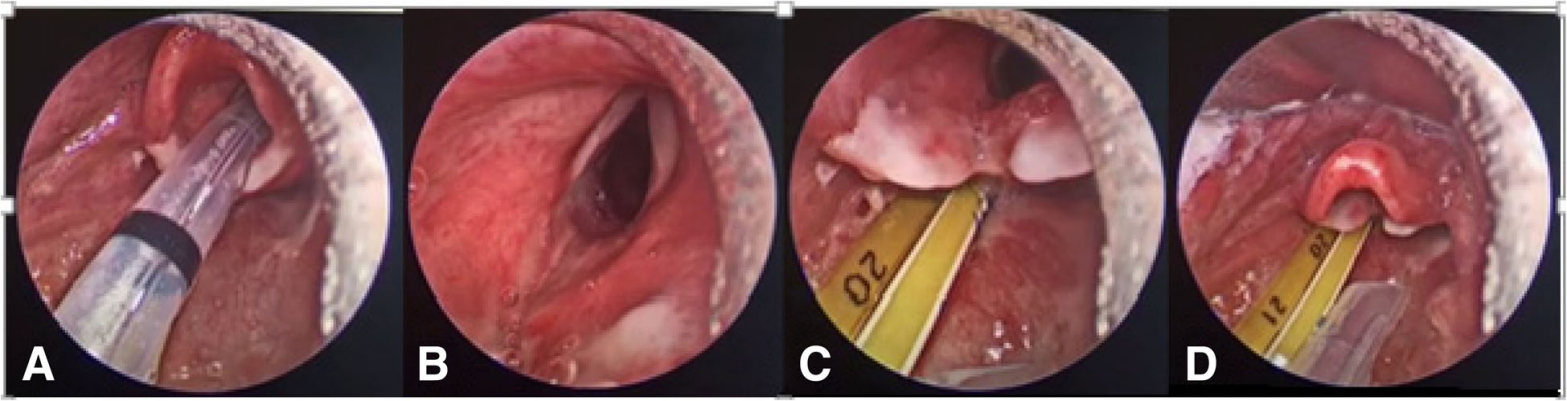
Fiberscopy 5 days post-burn showing (**a**): reduction of oedema; (**b**): restored airway; (**c**): pale arytenoid cartilage; **d**: thin epiglottis

Three days after initial intubation, the patient extubated himself. Because of a manifest inspiratory stridor and increased respiratory labour, he was reintubated via rigid fiberscopy in the operating theatre by an experienced otorhinolaryngologist. Rigid fiberscopy was his preferred method since he lacked experience with videolaryngoscopy. There was still evident swelling of the epiglottis (Fig. 2). Five days after intubation, fiberoptic laryngoscopy demonstrated a restored airway with a thin epiglottis, pale arytenoid cartilage (post-burn injury) and residual fibrinous coating in the postcricoid region, upon which the patient was successfully extubated (Fig. 3).

**Fig. 4:**
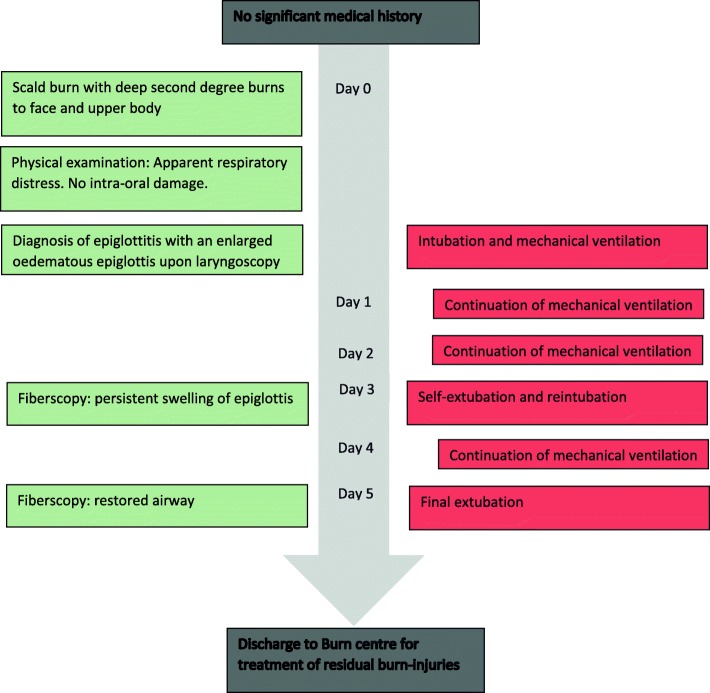
Timeline case report

For an overview of this case’s timeline, see Fig. 4.

## Discussion and conclusion

Airway damage following thermal injury is associated with inhalation of steam and heat due to hot liquid or fire [2, 3]. Incidents of burns after ingestion of hot beverages, food or objects resulting in injury of the respiratory tract have been reported [3, 4]. The mechanism of injury is consistent with the pattern of swallowing, also affecting the oral and gastro-intestinal tract. Lower airway burns are unusual due to heat conduction of pharynx and swallowing reflex [2, 5].

It is unusual for scalds to be accompanied by upper airway damage and obstruction, but several case-reports have emphasised it as a complication [2, 5, 7]. Thermal damage to the epiglottis after inhalation of large volumes of steam has been described in absence of oropharyngeal injury [8]. The time of onset of symptoms is variable from immediately after injury up to 72 h post-burn, warranting extended observation [5].

The type of scalding injury known as ‘teapot syndrome’, where hot liquid is grabbed by the child with the aim of ingestion and falls over a child causing burns on the face, upper thorax and arms, is known to cause peri-oral and facial oedema [3–5, 7, 8]. In such injuries, hot liquid may enter the oral cavity even in absence of ingestion, causing obvious intra-oral damage [8]. Additionally, hypopharyngeal damage should be expected, even though it rarely occurs in clinical practice in absence of ingestion. Although there was no intra-oral injury, this might be the mechanism of injury in the patient we have presented.

Thermal epiglottitis requires imminent airway protection. Literature suggests transportation of the patient to the operating theatre to create a maximally safe environment before airway manipulation [4, 8]. It must be noted that the time required for transportation delays intervention which might be disastrous for airway management. In our case in the Burn Centre, expertise and experience of staff and all equipment for management of a difficult airway is available in the emergency room. The initial management of a patient as described in this case is always done in a multdi-disciplinary setting in the presence of a burn physician, anaesthesiologist and paediatrician. In a regular emergency department, where all of these resources are not present, transportation might be beneficial. Given the evident stridor and respiratory distress the decision was made to instantly intubate upon which the clinical diagnosis was evident. No neck radiography was performed. Current practice is to not delay airway intervention by attempts to obtain radiography.

One could argue the HEMS-physician should have intubated upon primary assessment. Besides the obvious risks involved with out-of-hospital intubation of a child with epiglottitis, the physician reasoned the stridor to not be as evident as in the emergency room and particularly not progressive during primary survey and transportation. Furthermore, the child had a patent airway and no intraoral injuries or ingestion. Thermal epiglottitis was not among his differential diagnosis. This remark shows the importance of this case report: awareness of thermal epiglottitis might have resulted in an alternate approach at site of injury.

In conclusion, thermal epiglottitis following scalds to face, neck and thorax is rare and can occur even in absence of obvious ingestion of a damaging agent. Burns to the peri-oral area should raise suspicion of additional damage to oral cavity and supraglottic structures. Clinical signs of respiratory distress and stridor should be promptly evaluated to clinically diagnose impending upper airway obstruction requiring intubation.

## Abbreviations

HEMS: Helicopter Emergency Medical Services
TBSA: total body surface area
